# Supplementary material for: Accurate prediction of gene deletion phenotypes with Flux Cone Learning
Source: Nat Commun. 2025 Sep 26;16:8492. doi: 10.1038/s41467-025-63436-9 (PMC12475001; doi:10.1038/s41467-025-63436-9)
Supplement: Supplementary file 1 — Supplementary Information [file 41467_2025_63436_MOESM1_ESM.pdf]

SUPPLEMENTARY FIGURES AND TABLES

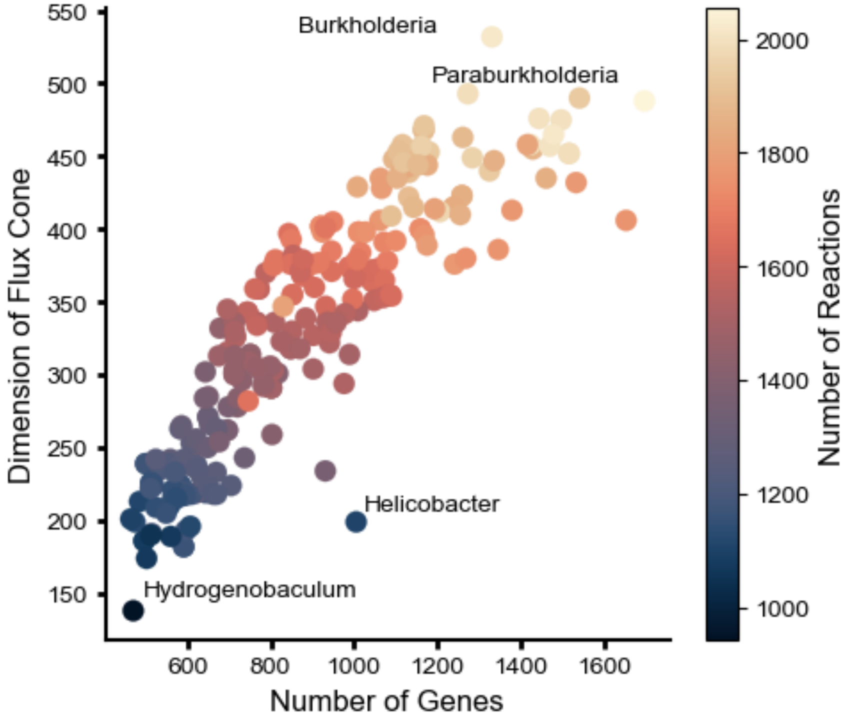

FIG. S1. Genome-scale metabolic models from across the bacterial kingdom. Models for $N = 244$ bacterial species were obtained from Plata et al, Nature, 2015 and species names were obtained from Ramon and Stelling, Nature Communications, 2023. The stoichometric matrices of each model were extracted, along with the number of genes (x-axis) and reactions (hue) in each model. The nullity of the stoichiometric matrix (the number of columns in the null space) was computed for each species (y-axis). Source data are provided as a Source Data file.

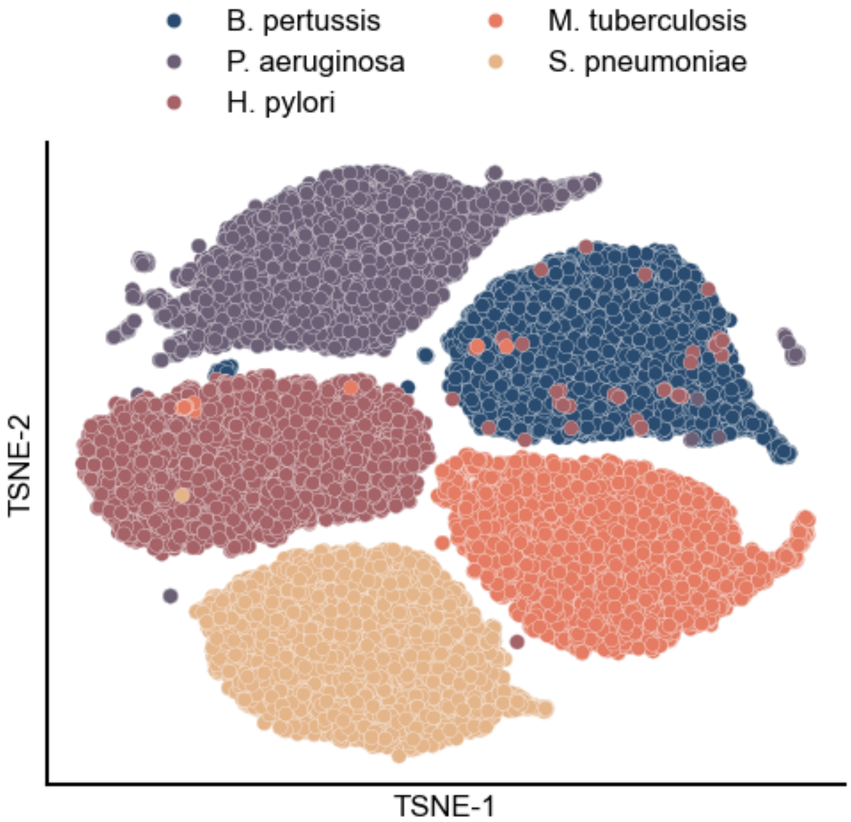

FIG. S2. Variational autoencoder compression of five bacterial pathogens. OptGPSampler was used to obtain $N = 5000$ samples ($k = 100$) from five bacterial GEMs selected from Plata et al, Nature, 2015. A variational autoencoder (VAE) was trained on the common $N = 494$ reactions shared across all species. The final embedding dimension D =8 was reached via 5 linearly decreasing ($D = 494$, $D = 397$, $D = 300$, $D = 203$, $D = 107$, $D = 8$) fully connected layers and the VAE was trained with PyTorch for 100 epochs with default hyperparameters using the Adam optimizer; t-SNE embeddings were employed for 2-dimensional visualization. The embeddings captures the separation of metabolic spaces across species. While the learned representation displays a small number of outliers, overall we found that all 5 pathogens retain distinct clustering despite the VAE only being trained on non species-specific reactions. Source data are provided as a Source Data file.

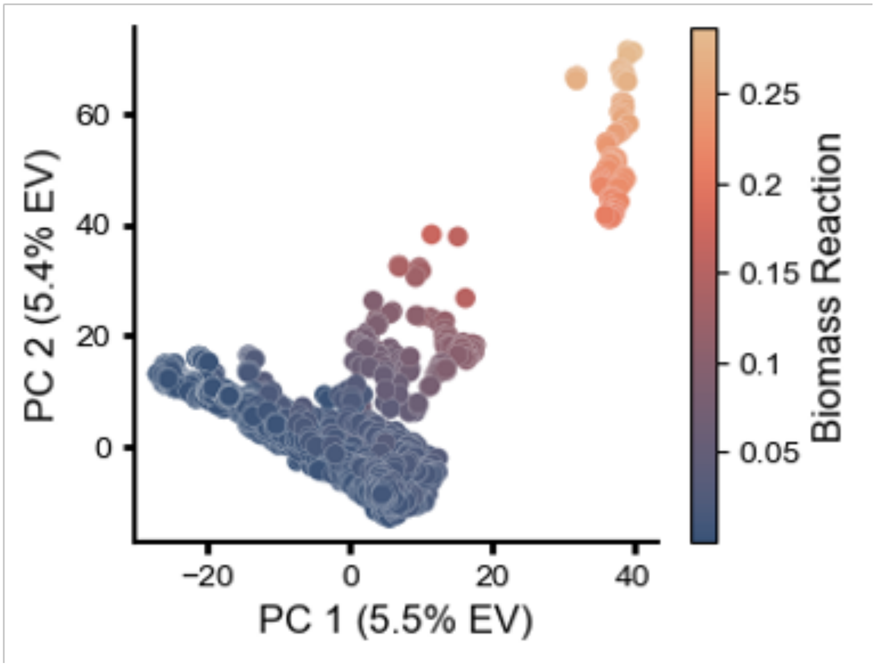

FIG. S3. PCA representation of flux cone of wild type *E. coli* metabolism. The unmodified (WT) iML1515 model was sampled 5000 times with a step size k=100.The resulting data was normalized using zero mean and unit variance and Principal Component Analysis (PCA) was performed. 621 PCs were required to explain 95% of variance in the scaled data. The first two PCs accounted for 5.5% and 5.4% of variance, respectively. The biomass reactions were removed from the PC calculations (see Supplementary Table S3) but samples on the plot are colored based on the value of the forward biomass reaction (BIOMASS_Ec_iML1515_core_75p37M). Source data are provided as a Source Data file.

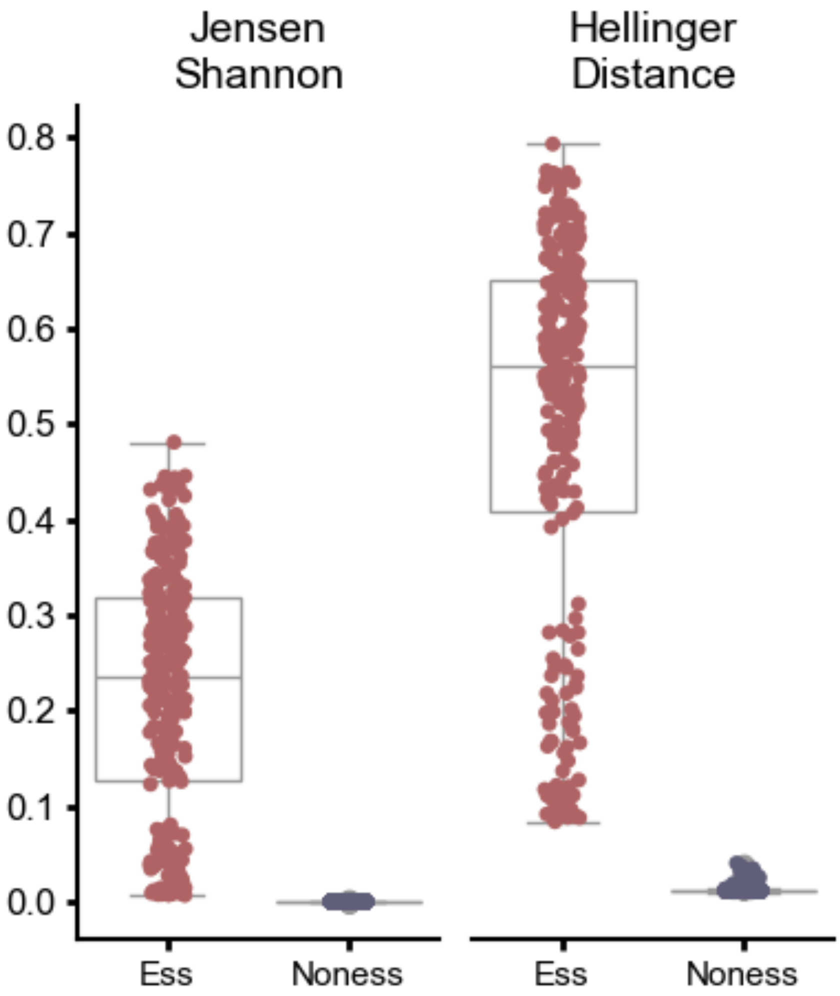

FIG. S4. Complete classifier sample scoring. We sought to exploit the excellent predictive performance of FCL to define a distance metric between deletions and the wild type strain. To this end, we retrained FCL on all *E. coli* gene deletions (N =1,502 genes), and computed the distribution of prediction scores for all flux samples in the wild type and each deletion strain. We then queried the model with flux samples of the wild-type GEM and each deletion cone (100 samples/cone), to produce distributions of prediction scores for the WT and each deletion strain. We scored each strain with the Jensen-Shannon divergence and Hellinger distance between the score distributions of each deletion and the wild type. We found statistically significant differences in score distribution of non-essential and essential genes, which reinforces the conclusion that perturbations to the flux cone are indicative of gene essentiality (one-sided t-test with $p = 7.46 \times 10^{-75}$ for JSD; $p = 5.47 \times 10^{-104}$ for Hellinger distance.). Source data are provided as a Source Data file.

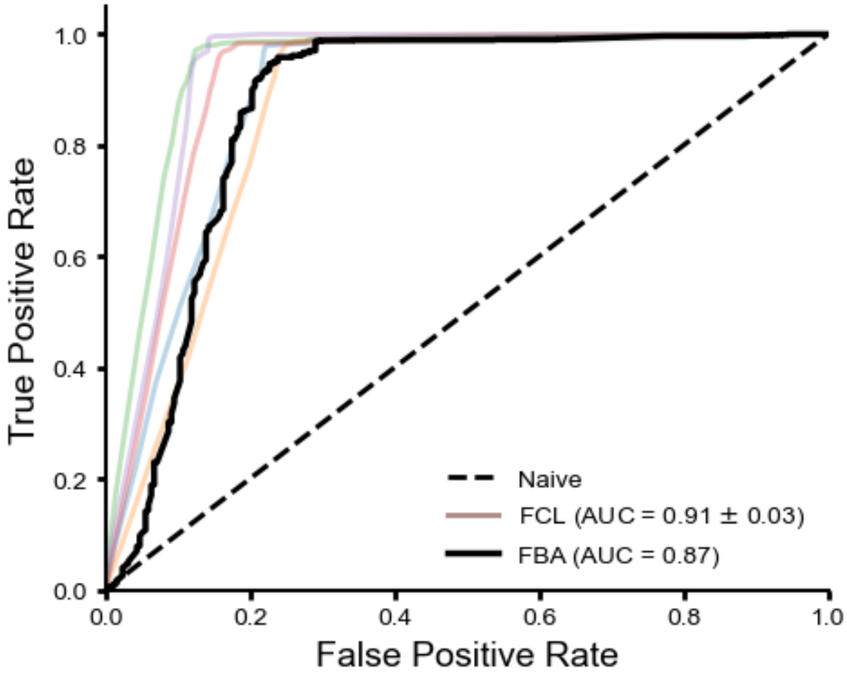

FIG. S5. Receiver operating characteristic (ROC) curves for *E. coli* essentiality prediction. FCL predictions were computed on a held-out set of test genes (20%) across five training repeats for a single model (100 samples/cone density) selected randomly from those in Figure 2A–B; FBA predictions were computed for all genes. Source data are provided as a Source Data file.

GEM	Number of genes	Number of reactions	Stoichiometric nullity
iML1515	1516	2712	867
iJO1366	1367	2583	817
iAF1260	1261	2382	752
ijr904	904	1075	332
Yeast9	1162	4130	1539
iCHO2291	2291	6236	2390

TABLE S1. Summary of genome-scale metabolic models employed in this study.

GEM	Sampled KOs	Total S	Density per cone	Step size	Subsampling	Size of NPZ file
iML1515	1502	150313	101	100	50	3.06Gb
iJO1366	1318	163680	124	5000	1	3.17Gb
iAF1260	1214	150784	124	5000	1	2.69Gb
ijr904	866	107508	124	5000	1	0.89Gb
Yeast9	1121	143716	124	1	1	4.43Gb
iCHO2291	2290	291028	127	1	1	13.53Gb

TABLE S2. Summary of model sampling details. All models were sampled with the same step size, except iML1515 which employed a smaller step size to generate more samples and the subsampled for the analysis in Figure 2.

GEM	Biomass Reactions Removed [IDs]
iML1515	BIOMASS_Ec_iML1515_core_75p37M [2669], BIOMASS_Ec_iML1515_core_75p37M_reverse_35685 [2670]
iJO1366	BIOMASS_Ec_iJO1366_core_53p95M [19], BIOMASS_Ec_iJO1366_core_53p95M_reverse_5c8b1 [14]
iAF1260	BIOMASS_Ec_iAF1260_core_59p81M [926]
ijr904	BIOMASS_Ecoli [269], BIOMASS_Ecoli_reverse_bf7a1 [270]
Total Common Reactions	864

TABLE S3. Biomass reactions removed for *E. coli* models and their common reactions.

Task	Class imbalance	Class breakdown
E. coli essentiality	83/17	N=1252 non-essential, N=251 essential
Yeast essentiality	86/14	N=964 non-essential, N=157 essential
CHO essentiality	83/17	N=1898 non-essential, N=392 essential
Yeast production	17/67/16	N=138 low, N=545 medium, N=128 high

TABLE S4. Class imbalances for each task.

Organism	Model	Accuracy	Precision	Recall	F1 Score	PR AUC	ROC AUC
Yeast	FBA	0.81	0.91	0.97	0.89	0.92	0.69
Yeast	FCL (5-fold CV)	0.90±0.008	0.91±0.008	0.98±0.010	0.94±0.007	0.94±0.021	0.78±0.065
Yeast	FCL (test)	0.89	0.89	0.99	0.94	0.93	0.73
CHO	FBA	0.85	0.86	0.97	0.91	0.86	0.62
CHO	FCL (5-fold CV)	0.85±0.014	0.86±0.010	0.97±0.008	0.91±0.008	0.91±0.029	0.72±0.068
CHO	FCL (test)	0.84	0.86	0.97	0.91	0.86	0.65

TABLE S5. Classification performance metrics for Flux Cone Learning (FCL) and Flux Balance Analysis (FBA) in yeast (*S. cerevisiae*) and Chinese Hamster Ovary (CHO) cells (Figure 3).

Model Name	Baseline	Resampled	Balanced	Both	Max % improvement
HistGradientBoostingClassifier	11.4	13.6	14.1	14.6	28.3
LinearSVC	23.8	24.0	27.2	26.8	27.2
LogisticRegression	23.3	26.2	29.5	28.9	14.2
RandomForestClassifier	18.1	11.7	19.1	18.4	5.5

TABLE S6. Accuracy for high producer deletion yeast deletion strains for each class balancing methods in Figure 4. Models were assessed across all genes in a held-out, class-stratified 20% test set (N =649 deletions).
